# Insights into the Overlap of Chronic Obstructive Pulmonary Disease and Sleep Apnea: Experience from the Clinic of Pneumology, Târgu Mureș

**DOI:** 10.3390/clinpract14060180

**Published:** 2024-10-29

**Authors:** Edith Simona Ianoși, Gall Zsuzsánna, Delia Rachiș, Dragoș Huțanu, Corina Budin, Paraschiva Postolache, Gabriela Jimborean

**Affiliations:** 1 Pulmonology Discipline, University of Medicine Pharmacy, Sciences and Technology “George Emil Palade”, 540139 Târgu Mureș, Romania; gabriela.jimborean@umfst.ro; 2 Pediatry Discipline, University of Medicine Pharmacy, Sciences and Technology “George Emil Palade”, 540139 Târgu Mureș, Romania; zsuzsanna.gall@umfst.ro; 3 Clinic of Pulmonology, Clinical County Hospital, 540011 Târgu Mureș, Romania; rachis.delia-liana.23@stud.umfst.ro (D.R.); hutanudragos1@gmail.com (D.H.); 4 Discipline of Pathophysiology, University of Medicine Pharmacy, Sciences and Technology “George Emil Palade”, 540139 Târgu Mureș, Romania; corina.budin@umfst.ro; 5 Department of Internal Medicine, Pulmonology and Clinical Pharmacology, University of Medicine and Pharmacy “Grigore T. Popa”, 700115 Iași, Romania; par.postololache@umfiasi.ro

**Keywords:** COPD, sleep apnea, overlap syndrome

## Abstract

Introduction: Chronic obstructive pulmonary disease (COPD) has a severe impact on patients’ health and can lead to multiple complications. Material and methods: We analyzed the co-occurrence of obstructive sleep apnea (OSA) in COPD patients hospitalized in the Pneumology Clinic of Târgu Mureș, Romania. Results: A total of 150 COPD patients were investigated by clinical examination, STOP-BANG and Epworth questionnaires, ventilatory polygraphy (PG), EKG, cardiac ultrasound, blood lipids, and sugar. Sixty-eight patients (45.3%) had OSA associated with COPD. A total of 61.7% were COPD gr. E, and 30.8% were gr. B. Frequently shown symptoms were snoring and nonrestorative sleep (100%), somnolence (73.5%), nocturnal awakenings (41.17%), morning headache (32.3%), and aggravated dyspnea. Types of OSA included obstructive (97.05%), central (2.5%), and associated obesity–hypoventilation (39.7%). A total of 76.4% were recently diagnosed with OSA. Men predominated at 70.5%, 76.4% were smokers, and 61.7% had experienced alcohol abuse. A total of 25% were overweight, and 71% had obesity. A total of 13.2% belonged to the category of 38–50-year-olds, 55.8% were in the 51–65-year-old category, 17.6% were in the 66–70-year-old category, and roughly 13.2% were in the 71-year-old category. Overlap syndrome (OS) comorbidities and complications were frequently present: 41% experienced respiratory failure, 66.1% experienced blood hypertension, 58.8% experienced ischemic cardiac disease, 32.35% experienced diabetes mellitus, 50% experienced dyslipidemia, and 29.4% experienced cor pulmonale. Conclusions: OS conferred gravity or directly contributed to cardiovascular, respiratory, and metabolic complications. OS was associated with more severe COPD and obesity. The prevalence of smoking in OS patients was higher than the national/European average.

## 1. Introduction

The association of sleep apnea (OSA) with other respiratory conditions like chronic obstructive pulmonary disease (COPD), asthma, bronchiectasis, fibrosis, silicosis, and cystic fibrosis, named “Overlap Syndrome” (OS), was identified almost 35 years ago in 1985 by David C. Flenley and initially represented an association of OSA in patients with COPD. Patients who share both disorders have flow limitation, on one hand, of the superior airways (OSA) and also flow limitation of the inferior airways, on the other hand (COPD) [1].

The high prevalence of OS (COPD and OSA), 10–30%, is explained by the high prevalence of both diseases in the general population and partially by common risk factors for both diseases—obesity, smoking, sedentary living, and advanced age [2,3]. Both COPD and OSA diseases have an onset age in adults around 45–60 years.

Isolated COPD is associated with various sleep and breathing disorders during sleep: insomnia (by cough, dyspnea), frequent awakenings (through coughing, obstruction, wheezing, adverse effects of medication), nocturnal hypoxemia (through hypoventilation, abnormalities of the ventilation/perfusion ratio), and an increased cardiovascular and metabolic risk. OSA is associated with nocturnal hypoxemia, an increase in sympathetic tone, sleep fragmentation, diurnal lack of energy, deconditioning to effort, etc. OSA is also considered an independent risk factor for cardiovascular, metabolic, or neuropsychiatric impairment [4].

COPD is very frequently associated with obstructive sleep apnea (OSA) but also with central sleep apnea (changes in the drive of the respiratory centers in severe COPD with cardiovascular complications and hypercapnia) [5].

Patients with OS experience more severe hypoxemia than patients with OSA or COPD alone [1]. OS can also be associated with respiratory-effort-related arousals (RERAs), which also cause sleep fragmentation and nocturnal awakenings [6,7]. At the same time, in OS associated with obesity, there are conditions for obesity–hypoventilation syndrome (OHS), which greatly aggravates the nocturnal hypoventilation existent in severe COPD. OHS is a common condition characterized by the presence of hypoventilation while the subject is awake, which is worse during the night, and is defined as the presence of obesity associated with daytime hypercapnia. The association of OHS could aggravate the already existing OS.

The combined effect of these conditions (COPD, OSA or CSA, and OHS) on ventilatory load, gas exchange, comorbidities, and quality of life is greater than either condition alone. OS will, thus, include serious clinical aspects, a more reserved prognosis, and an increased risk of mortality due to the association of respiratory, cardiovascular, metabolic, and neuropsychic complications from the two diseases.

In OS, compared to isolated OSA or COPD, there is more accentuated daytime sleepiness, more severe nocturnal desaturations, diurnal/nocturnal hypercapnia, pulmonary hypertension, and early chronic pulmonary heart disease (with mild/moderate pulmonary functional decline) [1].

Active search for OSA in COPD by polysomnography/polygraphy is recommended in the presence of specific OSA symptoms and risk factors and in the case of hypoxemia, polyglobulia, and cor pulmonalis (especially in COPD with a modest obstruction or with early cognitive decline). On the other hand, all patients with OSA will be investigated by respiratory function tests.

Due to the early complications, OS requires complex treatment (for both conditions) with the correction of OSA with permanent continuous airway pressure or bilevel airway pressure and with the association of oxygen therapy in patients who remain hypoxemic. Similarly, treatment of OSA in the overlap syndrome can favorably affect both OSA and COPD outcomes [8].

## 2. Materials and Methods

**Figure 1:**
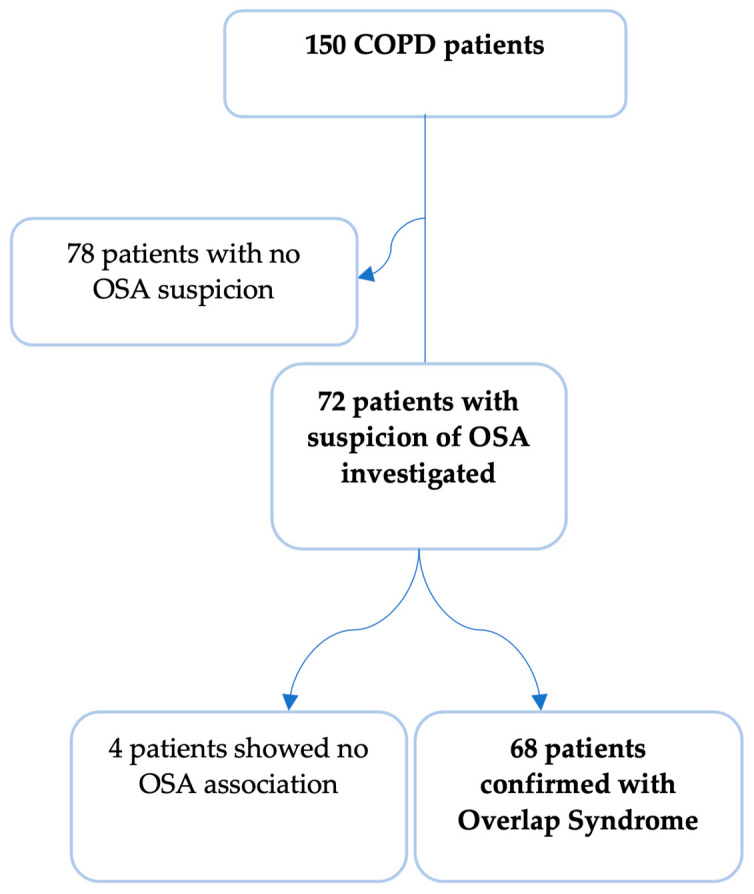
Patient selection process. Bold is used to emphasize the study population final selection process.

From 150 patients with COPD successively hospitalized in the Pulmonology Clinic in the period of 2023–2024, based on suspicion of the coexistence of OSA (respiratory deficiency (COPD) combined with suggestive anamnesis or heteroanamnesis for OSA—snoring, sleep-choking, nonrestorative sleep, daytime sleepiness), we selected 72 patients (Figure 1) in whom we performed targeted investigations for the diagnosis of breathing disorders during sleep: (a) careful clinical examination; (b) Epworth sleepiness questionnaire; (c) STOP-BANG risk questionnaire; (d) 6-channel ventilatory polygraphy (nasal flow, thoraco-abdominal exercise tape, pulse oximetry (SaO_2_, peripheral pulse rate), body position, snoring); and (e) investigations for the possible complications of SA: cardiological examination, blood pressure, EKG, blood tests for metabolic disorders (blood sugar, lipids, cholesterol, triglycerides). Body weight was evaluated in all patients through the body mass index evaluation (BMI), with grading being carried out as follows: BMI < 25—normal weight, BMI between 25 and 30—overweight, BMI between 30 and 35—grade I obesity, BMI between 35 and 40—grade II obesity, and BMI > 40—grade III obesity. OSA was characterized considering the apnea–hypopnea index (AHI) as follows: mild OSA with AHI < 15/h, moderate OSA with AHI between 15/h and 30/h, and severe OSA with AHI > 30/h. Epworth and STOP-BANG scales were used and interpreted as follows: Epworth scale of 10–15: moderate sleepiness, Epworth scale 16 or higher: accentuated sleepiness, STOP-BANG score of 3–4: moderate risk of OSA, and STOP-BANG score of 5–8: high risk of OSA.

For statistical analysis and comparison of the data, the two-sided Fisher exact test and chi-square test were used to calculate the odds ratio (OR) risk using IBM SSPS Statistics 26.0.0.0 software. Differences were considered statistically significant at *p* < 0.05 using 95% confidence intervals.

## 3. Results

In total, 68 (94.4%) out of 72 patients with suspicion to have overlap syndrome OS (COPD and sleep apnea) were confirmed by clinical investigation, questionnaire, and polygraphy (PG), which showed the correctness of the assumption. OSA was detected in 68/150 of the total group of COPD patients randomly selected—45.3%. In this subgroup, the severity of the COPD was established upon several criteria (upon the GOLD guide 2024):

(a) Symptoms—CAT (COPD assessment test): <10—gr. of risk A, >10—gr. of risk B.
(b) Number of exacerbations in the previous year: ≥2 moderate exacerbation or 1 severe with hospitalization = gr. of risk E.
(c) Presence of comorbidities.
(d) Spirometry quantified the severity of airflow obstruction by analyzing the main parameter (FEV1).
  In our study, patients with an advanced disease predominated with group E identified in 61.7% of patients, with multiple exacerbations and comorbidities.

Men predominated in the study population with 48 (70.5%). Among risk factors for OSA we discovered high percentages of smoking (76.4%; 40 males and 12 women), professional exposure to noxious air (60.29%), and daily alcohol consumption (58.8%) (Table 1).

From the 52 patients with affirmative smoker status, 32 (61.5%) were former smokers but all heavy ex-smokers (at least 25 packs/year). The 8 nonsmoker women previously had secondhand exposure to cigarette smoking and also intense exposure to chemical substances (fixatives in hairdressing services and exposure to gases resulting from home biomass burning).

OSA was in great prevalence in active people between 51 and 65 years old (55.8%), especially in men this age (70.83%), and 13.2% in young men 38–50 years old (Table 2).

Symptoms in our group were collected from the patients and with the help of the patients‘ bed partner. They were multiple and partially covered by the symptoms of the COPD itself (Table 3). Symptoms were more frequent in the advanced stages of COPD.

Two symptoms were present in all the patients and need to be searched in any patient with COPD: snoring and nonrestorative sleep. In fact, the CAT questionnaire also questions sleep disorders. OS-associated multiple symptoms originate from both diseases but also from the aggravation of complications (Table 3).

In our group, obstructive apnea predominated (97.05%), followed by central apnea (2.94%), while 39.7% of patients had concomitant obesity–hypoventilation syndrome (Table 4).

Concomitant obesity–hypoventilation syndrome (OHS) diagnosis was established under multiple criteria:

(a) Exaggerated dyspnea on exertion.
(b) Severe obesity, mainly troncular/abdominal.
(c) Facial plethora (polycythemia and hypercapnia by vasodilation).
(d) Awake room air peripheral saturation (SpO_2_) ≤ 94 percent or an overnight nadir saturation < 80 percent.
(e) High desaturation on polygraphy (under 90%–around 30% from the night).
(f) Raised bicarbonate over 26 mmol/L or base excess >3 mmol/L in the absence of another cause for a metabolic alkalosis in an obese individual.

Sleep apnea and OHS were found in 27 patients from the 68 group—39.7% of patients with significant difference between genders (*p* < 0.001). Male patients were at higher risk of developing associated OHS (OR = 11.84, *p* < 0.001).

OS comorbidities were frequent in our group and are represented in Table 5.

The confirmation of cor pulmonale was established in the cardiology ward under the suspicion that we had upon the symptoms of pulmonary hypertension (elevated jugular pressure, hepatomegaly, +/− peripheral oedema) and ECG and upon right-sided heart failure by cardiac ultrasound.

The association with the overlap of COPD + OSA of OHS brought additional elements of severity and multiple comorbidities (Table 5).

From the group, 10 patients (all with triple overlap COPD, OSA, and OHS) necessitated treatment with noninvasive ventilation, and 6 were transferred in ICUs. Fatality was high in these patients, at 50%.

During initial investigations through first-hand questionnaires (Table 6), patients predominantly presented high risk of OSA development (through STOP-BANG scores higher than 4 points), with males at 91.66% and females at 80%, while also presenting high prevalence of daytime sleepiness (through Epworth scale scores higher than 10), with males at 85.41% and females at 90%. No significant differences were noted between genders.

After polygraphy (Table 7), all patients presented at least moderate OSA (AHI > 15 events/hour), with 14.70% having moderate OSA and 85.30% having severe OSA, with AHI values higher than 30 events/hour. However, all patients diagnosed with associated OHS present severe forms of OSA, with AHI values higher than 30 events per hour.

Treatment of OS was started with general measures on patient education and lifestyle changes: smoking and alcohol cessation, weight loss in obese patients, and increase in physical activity. All COPD patients underwent an increase in treatment with inhaled bronchodilators (antimuscarinic agents for group A COPD and a combination of long-acting beta-agonists plus antimuscarinic agents in group B and triple therapy (with association of inhaled corticosteroids in group E)).

Treatment with devices with continuous positive airways pressure (CPAP) was the main recommendation for OS, to avoid worsening of the symptoms/complications, along with smoking cessation and, for future weight loss, bariatric surgery in selected patients.

Unfortunately, only 51 from 68 initially accepted CPAP usage during sleep despite the repeated medical recommendations (compliance 75%). Five patients with severe obesity–hypoventilation syndrome benefited from home nocturnal noninvasive ventilation.

In patients that remained hypoxemic after CPAP, oxygen therapy was introduced during sleep, associated with PAP therapy.

Twelve patients were subsequently readmitted after discharge for aggravated symptoms or for re-evaluation of the treatment (after an interval of time between 2 and 6 months). Those patients were referred by the specialists from internal medicine, cardiology, or general surgery (before bariatric surgery within the preanesthetic and preoperative balance). All those 12 patients finally accepted the treatment with CPAP.

## 4. Discussion

Many respiratory diseases can be associated with OSA: COPD, asthma, bronchiectasis, mucoviscidosis, and/or diffuse fibrosis. The most studied association is the so-called COPD–OSA overlap. The high prevalence of OS (more than 10% of those with COPD) is explained by the high prevalence of both diseases but also due to common etiopathogenetic conditions (smoking, exposure to noxious substances) [2,9]. The conditions leading to a diagnosis of COPD are very frequently met, and it is currently in third place in terms of all causes of mortality in the world; in the USA, it is the cause of 4% of all deaths [9]. The prevalence of OSA alone in the United States is approximately 30% [10,11]. Shawon et al. found a concomitance of COPD and OSA between 2.9% and 65.9%, especially in moderate or severe COPD [11].

In our study, we found OSA in 45.3% of the patients with COPD hospitalized in condition of high prevalence of intricated risk factors: obesity, alcohol consumption, smoking, and exposure to pollutants. A total of 61.7% of patients with OS were in the advanced group of COPD upon OLD classification.

OHS usually is associated with OSA in obese people (up to 90%) and increases the risk for pathologic breathing also on the lower respiratory tract, especially during sleep by hypoventilation [12,13]. OHS is defined by (a) increased body mass index (BMI) over 30 kg/m^2^ associated with diurnal hypoventilation; (b) increased partial pressure of carbon dioxide (PCO_2_) ≥ 45 mmHg (in blood gas analysis or measured by transcutaneous PCO_2_); (c) hypersomnolence; and if it is associated with OSA, loud snoring, choking during sleep, fatigue, and neuropsychic decline [13].

We found OHS predominantly in men (52%) compared with 10% in women. Data from the literature suggest a lower prevalence in women [14]. From patients with OHS, about 33% have acute or chronic respiratory failure [15]. In our study, because of the complex overlap of COPD, OSA, and OHS, we found a huge percentage of patients with respiratory chronic failure (92.59%).

Our patients with OS had common risk factors—smoking, exposure to noxious substances, and alcohol abuse in a significant proportion compared with the general population. For example, smoking had a huge average value in our patients (76.4% (more in men) over the national and European values (28% versus 19.7% of the EU population)).

Men predominated, with 70.5% in our study. Among risk factors for OSA, we discovered high percentages of smoking 76.4% (40 males and 12 women), professional exposure to noxious air (60.29%) (biomass, fixative from hairdressing, pesticides, diluents in leather industry), and daily alcohol consumption (58.8%). Smoking and exposure to noxious particles determined chronic inflammation both in the upper airways (favoring OSA) and in the lower airways (determining COPD). At the same time, the effects of smoking and noxious substances are carried out at the level of the cardiovascular system [16] and also increase the risk for lung and laryngeal cancer [17].

Another risk factor contributing to the occurrence of the two severe conditions in OS is air pollution, especially in an industrial city such as Târgu Mures [18].

Obesity was a common risk factor for sleep apnea, for OSH, but also for worsening respiratory, cardiovascular, and metabolic comorbidities [19,20,21,22]. In patients with milder disease, mortality may rise with BMI > 30 kg/m^2^ [23] by reduction in fat-free tissue and by a sedentary lifestyle [24].

At the same time, it is known that obesity causes important effects on respiratory function—restrictive dysfunction and decreased compliance, by favoring diabetes and diabetic pneumopathy [24,25]. Patients with obesity also have a complex metabolic impairment—metabolic syndrome, increased risk of diabetes, dyslipidemia [25], and an increased risk of unfavorable evolution in patients with advanced COPD [26]. Therefore, the fact that patients with OS have an increased risk is justified.

In our study, conditions for a diagnosis of obesity were frequently met (72.5%), especially second-degree and third-degree obesity (17.6%), with eight patients being over 150 kgs and four over 120 kgs. Advanced obesity was more frequent in women (70%) compared to men (49.16%) (Table 1).

Unfortunately, the insurance coverage for bariatric surgery is not well defined, and there are not enough centers for obesity counseling and treatment.

Patients with OS have increased risk of nocturnal hypoxemia as compared to COPD patients, resulting in respiratory failure and hypercapnia and risk for central sleep apnea by disorders at the level of the central respiratory centers. Underlying hypoxemia in OS (originating from OSA, obesity, and COPD) induces the release of systemic inflammatory mediators including C-reactive protein, interleukin-6 and 8, tumor necrosis factor-alpha, and reactive oxygen species [27,28,29].

We found an increased number of complications in our study, directly caused or aggravated by both diseases (COPD and OSA) (Table 5): respiratory failure, systemic hypertension, ischemic cardiac disease, arrhythmias, cor pulmonalis, diabetes, dyslipidemia, cognitive decline, and respiratory infections. All complications were very frequently in the complex overlap of COPD plus OSA together with obesity–hypoventilation syndrome.

Isolated COPD is associated with various sleep and breathing disorders during sleep: insomnia, frequent awakenings (through coughing, obstruction, wheezing, adverse effects of medication) [6], nocturnal hypoxemia (through hypoventilation, abnormalities of the V/Q ratio), and an increased cardiovascular and metabolic risk [22]. At the same time, COPD has direct effects for increasing OSA intensity, by sleep quality reduction, oxygen desaturation during sleep, rostral fluid shift in supine position, and even through the effects of corticoid therapy [12].

OSA and OHS alone are independent risk factors for cardiovascular, metabolic, or neuropsychiatric impairment [2,4,12,15,30]. Long night periodical hypoxia episodes determine inflammation with oxidative stress and release of systemic inflammatory mediators like TNF-α, IL-6, IL-8, and CRP, which lead to endothelial dysfunction and atherosclerotic plaque formation [27,28]. At the same time, OSA and OHS generate metabolic dysfunction including insulin resistance and abnormal lipid metabolism, being associated frequently with systemic hypertension, coronary heart disease, cardiac failure, arrhythmias, and stroke [28,31,32].

Patients with OS have more cardiovascular comorbidities and an increased risk of pulmonary hypertension and right heart failure, secondary to hypoxemia and long-term-untreated hypercapnia compared to patients with isolated COPD or OSA [33,34,35]. Hawrylkiewicz et al. observed in their study that 86% of the subjects with OS had pulmonary hypertension as compared to 16% of subjects with OSA alone [33,34,35]. OS is also associated with an increased risk of cardiac rhythm disorders [34,35,36].

Our patients had several comorbidities and complications (being greater in advanced COPD).

OS will, thus, include serious clinical aspects, more reserved prognosis, and increased risk of mortality due to the association of respiratory, cardiovascular, and metabolic complications from the two diseases [1,2]. In OS, compared to isolated OSA or COPD, more pronounced daytime sleepiness, more severe nocturnal desaturations, diurnal/nocturnal hypercapnia, pulmonary hypertension, and early chronic pulmonary heart disease (with mild/moderate pulmonary functional decline) are noted [1,2,19,21,31].

Diabetes mellitus (DM) had a high proportion in OS patients, compared to the general population, of the risk that emerged from both diseases [1,37,38,39,40,41]. The relation between DM and OSA is well known. DM is associated more frequently with OSA through neuropathy and impaired central control of breathing, deficient neural reflexes related to the upper airways, obesity, and by cardiovascular complications that aggravate OSA [38]. OSA causes an increased risk of insulin resistance diabetes and β-cell dysfunction (sympathetic activation, oxidative stress, systemic inflammation, adipokine dysregulation, obesity, and diurnal hypersomnolence) [40,41]. At the same time, COPD increases the risk for DM by systemic inflammation, influence of smoking on genes linked to diabetes and COPD [37], preexisting conditions, lifestyle, and weight gain [41].

We also noticed an increased risk for respiratory infection and COPD exacerbation (≥2/year) in patients with OS. This observation is consistent with the data from the literature and is partially explained by intermittent hypoxia during OSA episodes which impairs local immunity at the pulmonary parenchymal level, by chronic local inflammation and damage, along with vascular endothelial dysfunction, resulting in an increased risk for infection and severity, even enhancing infections, including the one with SARS-CoV-2 [42,43,44,45,46,47].

The frequently met DM in both COPD and OSA patients also increases the risk for super-infection [48]. In COPD, chronic inflammation compromises innate and adaptive immune responses and facilitates recurrent respiratory infections and exacerbations [45,46]. Those at risk of developing infections and bad evolution prognosis were also patients with associated obesity withing the OS (known as a risk factor for impaired immune function). OS was also associated with increased mortality [49]. In this study, within patients with CPAP or NIV treatment, we did not record fatalities.

In such conditions of severity with regard to the symptoms and complications in OS, the treatment has to be complex and multimodal [1,11,13,15,21,50,51,52].

Lifestyle change was the first general measure recommended in our study in all patients: increased general and organized physical activity, pulmonary rehabilitation (PR), and diet with calories/sugar reduction for weight loss. PR is recognized to bring benefits at the same time in OSA and COPD (where skeletal muscle wasting could be present), improvement in AHI and sleep quality, optimizing mood and dyspnea score, combating sedentarism [50,51].

Correct treatment of COPD with dual bronchodilators or triple therapy in severe COPD with exacerbation (highlighted by increased number of eosinophils) may improve sleep quality and REM sleep in moderate/severe COPD. It is not very clear if OSA will also be improved but it is known that treatment with bronchodilators ameliorates dyspnea, and patients with COPD can better perform pulmonary rehabilitation.

Bronchodilators (especially long-acting bronchodilators and combining antimuscarinic with beta 2 sympathomimetic agonists) are the central treatment of COPD by multiple effects (GOLD COPD 2024) [52]: they (a) alleviate bronchial obstruction, airflow limitation, and symptoms; (b) reduce hyperinflation; (c) improve exercise tolerance and permit easy performance of pulmonary rehabilitation; (d) improve lung function [52]; (e) improve quality of life [53]; (f) reduce the use of crisis medication [53]; (g) reduce exacerbations; and (h) improve survival along with association of inhaled corticoids/oxygen or noninvasive ventilation in hypercapnic patients.

Therapy of the overlap syndrome consists of treatment with devices that ensure a positive airways pressure (PAP) in the airways in continuous mode during inspiration and expiration (CPAP) or with different inspiratory and expiratory pressures (bilevel PAP) or by nocturnal noninvasive ventilation (NIV) in cases with OS and established hypercapnia/hypoventilation with or without associated nocturnal O_2_. Several studies have shown that PAP therapy in OS reduces daytime and nocturnal hypercapnia, improves nocturnal hypoxemia, lowers the apnea–hypopnea index, decreases the pulmonary hypertension, decreases mortality and exacerbation in OS, and improves sleep quality and symptoms (diurnal sleepiness) [13,21,54,55,56,57,58,59,60,61,62,63]. Other studies show the specific decrease in the inflammatory markers during treatment with CPAP in OS [64,65].

NIV devices are also indicated in COPD exacerbation and acute respiratory failure [50]. They also help to improve respiratory muscle strength and endurance in these patients by reducing the workload of breathing. When comparing the use of CPAP or NIV, both methods of treatment were equally effective in improving daytime PaCO2 and sleepiness (especially in OHS) [66].

## 5. Conclusions

Overlap syndrome (COPD and OSA) was frequently met in our hospitalized patients in the Clinic of Pneumology of Târgu Mureș, Romania. Polygraphy (PG) was a comfortable method for efficient and rapid diagnosis of associated OSA and COPD. The high positivity of PG for OSA detection (94.4%) in suspected patients proved the correct suspicion harbored by clinical symptoms, questionnaires (Epworth sleepiness questionnaire and STOP-BANG), and close physical examination. OS presented multiple intricated risk factors for both diseases. The prevalence of smoking in OS patients was higher than the national/European average.

Males predominated in our study, especially from active socioprofessional groups. OSA was in great prevalence in active people between 51 and 65 years old, including young men (38–50 years old). OS was associated with multiple symptoms, originating from both diseases but also from their complications. Symptoms were more frequent in the advanced stages of COPD. OS was associated with more severe COPD and advanced obesity.

The great number of newly diagnosed OSA and OHS and the severity of OS indicated the late diagnosis, and invited us to highlight this simple message: OSA and comorbidities (cardiovascular, metabolic, cognitive decline) should be actively screened in all COPD patients through clinical examination, sleep questionnaires, PG, and multidisciplinary consultations; at the same time, all patients with OSA will have respiratory functional tests, conducted for lung function evaluation (COPD, asthma, or restrictive diseases) to eliminate or, depending upon the case, to detect and treat a concurrent disease in the lower airways as early as possible.

OS conferred gravity or directly contributed to cardiovascular, respiratory, and metabolic complications emanating from both diseases (COPD and OSA). Given the severe complications, OS requires increased treatment for both diseases (COPD and OSA). The main treatment for OSA will be CPAP for upper airway obstruction and NIV for hypoventilation, with oxygen therapy given to remaining hypoxemic patients.

## Figures and Tables

**Table 1:** OS risk factors were risk factors for both OSA and COPD.

	Total Patients n = 68	Females n = 20	Males n = 48	*p*/OR (CI 95%)
Smoking	52 (76.4%)	12 (60%)	40 (83.3%)	0.04/3.33 (1.03–10.77)
Ambiental and professional exposure (pesticides, dilutant in leather industry, biomass burning, second hand smoking, exhaust gases)	41 (60.29%)	8 (40%)	33 (68.7%)	0.03/3.30 (1.11–9.74)
Alcohol consumption	42 (61.7%)	4 (20%)	38 (55.8%)	**<0.001/15.20 (4.14–55.68)**
Overweight	17 (25%)	4 (20%)	13 (27%)	0.54/1.48 (0.41–5.27)
Obesity	49 (72.05%)			
Obesity degree 1	11 (16.17%)	2 (10%)	9 (13.23%)	0.10
Obesity degree 2	26 (38.2%)	12 (60%)	14 (29.16%)	
Obesity degree 3	12 (17.6%)	2 (10%)	10 (20.8%)	

Bolded values are statistically significant (*p* < 0.05).

**Table 2:** Distribution of overlap syndrome upon age.

	38–50 Years Old	51–65 Years Old	66–70 Years Old	Over 71 Years Old	*p*
**Women**	2 (10%)	4 (20%)	8 (40%)	6 (30%)	**<0.001**
**Males**	7 (14.58%)	34 (70.83%)	4 (8.33%)	3 (6.25%)	
Total	9 (13.2%)	38 (55.8%)	12 (17.6%)	9 (13.2%)	

Bolded values are statistically significant (*p* < 0.05).

**Table 3:** Symptoms related to group of COPD severity.

	Total Patients n = 68	COPD gr. of Risk A n = 5	COPD gr of Risk B n = 21	COPD gr. of Risk E n = 42	*p*
Snoring	68 (100%)	5 (100%)	21 (100%)	42 (100%)	1.00
Nonrestorative sleep	68 (100%)	5 (100%)	21 (100%)	42 (100%)	1.00
Awakening with gasping or choking	28 (41.17%)	0 (0%)	8 (38.09%)	20 (47.61%)	0.43
Nocturnal dyspnea	24 (35.29%)	0 (0%)	3 (14.2%)	21 (50%)	**0.01**
Morning headaches	22 (32.3%)	0 (0%)	5 (23.80%)	17 (40.47%)	0.33
Daytime sleepiness or fatigue,	50 (73.5%)	2 (40%	15 (71.42%)	33 (78.57%)	0.17
Poor concentration or memory impairment	59 (86.7%)	5 (100%)	20 (95.23%)	34 (80.95%)	0.30

Bolded values are statistically significant (*p* < 0.05).

**Table 4:** Respiratory sleep disorder diagnosed in patients with COPD.

	Obstructive Sleep Apnea or Mixt Sleep Apnea	Central Sleep Apnea	Associated Obesity–Hypoventilation Syndrome	*p* for OHS/OR (CI95%)
Women n = 20	20 (100%)	0 (0%)	2 (10%)	**<0.001/11.84 (2.44–57.37)**
Males n = 48	46 (95.83%)	2 (4.16%)	25 (52.03%)	
Total n = 68	66 (97.05%)	2 (2.94%)	27 (39.7%)	

Bolded values are statistically significant (*p* < 0.05).

**Table 5:** Relevant comorbidities for patients with overlap syndrome (COPD + OSA + OHS).

	Total n = 68	COPD + OSA Without OHS n = 41	COPD + OSA + OHS n = 27	*p*/OR (CI 95%)
Respiratory failure	28 (41%)	3 (7.31%)	25 (92.59%)	**<0.0001**/158.30 (24.67–1016.13)
Respiratory failure + NIV need (6 in ICU)	10 (14.7%)	2 (4.87%)	8 (29.62%)	**0.01**/8.21 (1.58–42.48)
Systemic hypertension	63 (92.64%)	38 (92.68%)	25 (92.59%)	0.98/0.98 (0.15–6.33)
Ischemic cardiac disease	40 (58.8%)	20 (48.78%)	20 (74.07%)	**0.04**/3.00 (1.04–8.62)
Arrythmia	14 (20.58%)	8 (19.5%)	12 (44.4%)	**0.03**/3.30 (1.11–9.74)
Cor pulmonale	30 (44.11%)	13 (31.7%)	17 (62.96%)	**0.01**/3.66 (1.31–10.16)
Diabetes mellitus	29 (42.64%)	14 (34.1%)	13 (48.1%)	0.25/1.79 (0.66–4.83)
Dyslipidemia	44 (64.7%)	21 (51.21%)	23 (85.18%)	**<0.01**/5.47 (1.60–18.65)
Increased risk for respiratory infection and COPD exacerbation (≥2/year)	40 (58.8%)	21 (51.21%)	19 (70.37%)	0.11/2.26 (0.80–6.32)
Cognitive decline: loss of concentration, mood swings, memory troubles, insomnia	48 (70.5%)	23 (56%)	25 (92.59%)	**<0.01**/10.32 (2.16–49.29)

Bolded values are statistically significant (*p* < 0.05).

**Table 6:** Questionnaire investigation results.

	Males n = 48	Females n = 20	*p*/OR (CI 95%)
Epworth scale score ≥ 10 pts	41 (85.41%)	18 (90%)	0.61/0.65 (0.12–3.44)
STOP-BANG scale score > 4 pts	44 (91.66%)	16 (80%)	0.18/2.75 (0.61–12.31)

Bolded values are statistically significant (*p* < 0.05).

**Table 7:** Polygraphy investigation results.

	Mild OSA (AHI < 15)	Moderate OSA (15 ≤ AHI ≤ 30)	Severe OSA (AHI < 30)
Total patients n = 68	-	10 (14.70%)	58 (85.30%)
Obesity–hypoventilation syndrome n = 27	-	-	27 (100%)

Bolded values are statistically significant (*p* < 0.05).

## Data Availability

The dataset is available on request from the authors.

## References

[1] Singh S., Kaur H., Singh S., Khawaja I. (2018). The Overlap Syndrome. Cureus.

[2] Budhiraja R., Siddiqi T.A., Quan S.F. (2015). Sleep disorders in chronic obstructive pulmonary disease: Etiology, impact, and management. J. Clin. Sleep Med..

[3] Heinzer R., Vat S., Marques-Vidal P., Marti-Soler H., Andries D., Tobback N., Mooser V., Preisig M., Malhotra A., Waeber G. (2015). Prevalence of sleep-disordered breathing in the general population: The HypnoLaus study. Lancet Respir. Med..

[4] Peker Y., Akdeniz B., Altay S., Balcan B., Başaran Ö., Baysal E., Çelik A., Dursunoğlu D., Dursunoğlu N., Fırat S. (2023). Obstructive Sleep Apnea and Cardiovascular Disease: Where Do We Stand?. Anatol. J. Cardiol..

[5] Kwon J.S., Wolfe L.F., Lu B.S., Kalhan R. (2009). Hyperinflation is associated with lower sleep efficiency in COPD with co-existent obstructive sleep apnea. J. Chronic Obstr. Pulm. Dis..

[6] Fleetham J., West P., Mezon B., Conway W., Roth T., Kryger M. (1982). Sleep, arousals, and oxygen desaturation in chronic obstructive pulmonary disease. The effect of oxygen therapy. Am. Rev. Respir. Dis..

[7] Omachi T.A., Blanc P.D., Claman D.M., Chen H., Yelin E.H., Julian L., Katz P.P. (2012). Disturbed sleep among COPD patients is longitudinally associated with mortality and adverse COPD outcomes. Sleep Med..

[8] Berry R.B., Kryger M., Roth T., William C., Dement W.C. (2017). Chapter 107—Sleep Related Breathing Disorders: Classification. Principles and Practice of Sleep Medicine.

[9] Pleis J.R., Ward B.W., Lucas J.W. (2010). Summary health statistics for U.S. adults: National Health Interview Survey, 2009. Vital Health Stat..

[10] Thornton A.T., Singh P., Ruehland W.R., Rochford P.D. (2012). AASM criteria for scoring respiratory events: Interaction between apnea sensor and hypopnea definition. Sleep.

[11] Shawon M.S., Perret J.L., Senaratna C.V., Lodge C., Hamilton G.S., Dharmage S.C. (2017). Current evidence on prevalence and clinical outcomes of co-morbid obstructive sleep apnea and chronic obstructive pulmonary disease: A systematic review. Sleep Med. Rev..

[12] Mokhlesi B. (2010). Obesity hypoventilation syndrome: A state-of-the-art review. Respir. Care.

[13] Masa J.F., Corral J., Alonso M.L., Ordax E., Troncoso M.F., Gonzalez M., Lopez-Martínez S., Marin J.M., Marti S., Díaz-Cambriles T. (2015). Efficacy of Different Treatment Alternatives for Obesity Hypoventilation Syndrome. Pickwick Study. Am. J. Respir. Crit. Care Med..

[14] BaHammam A.S., Pandi-Perumal S.R., Piper A., Bahammam S.A., Almeneessier A.S., Olaish A.H., Javaheri S. (2016). Gender differences in patients with obesity hypoventilation syndrome. J. Sleep Res..

[15] Olson A.L., Zwillich C. (2005). The obesity hypoventilation syndrome. Am. J. Med..

[16] Yanbaeva D.G., Dentener M.A., Creutzberg E.C., Wesseling G., Wouters E.F. (2007). Systemic effects of smoking. Chest.

[17] Kiss B., Neagos C.M., Jimborean G., Sárközi H.K., Szathmary M., Neagos A. (2023). Comorbidities and Laryngeal Cancer in Patients with Obstructive Sleep Apnea: A Review. Medicina.

[18] Ianoși E.S., Jimborean G., Rachiș D.L., Jimborean O., Sárközi H.K., Gârbovan C., Vultur M.A. (2023). Global pollution—A public health problem the effects of pollution on the respiratory system. Proc. Rom. Acad. Ser. B.

[19] Poulain M., Doucet M., Major G.C., Drapeau V., Sériès F., Boulet L.-P., Tremblay A., Maltais F. (2006). The effect of obesity on chronic respiratory diseases: Pathophysiology and therapeutic strategies. CMAJ.

[20] McNicholas W.T. (2016). Chronic obstructive pulmonary disease and obstructive sleep apnea-the overlap syndrome. J. Thorac. Dis..

[21] Marin J.M., Soriano J.B., Carrizo S.J., Boldova A., Celli B.R. (2010). Outcomes in patients with chronic obstructive pulmonary disease and obstructive sleep apnea: The overlap syndrome. Am. J. Respir. Crit. Care Med..

[22] Sin D.D., Man S.F.P. (2003). Why are patients with chronic obstructive pulmonary disease at increased risk of cardiovascular diseases? The potential role of systemic inflammation in chronic obstructive pulmonary disease. Circulation.

[23] Landbo C., Prescott E., Lange P., Vestbo J., Almdal T.P. (1999). Prognostic value of nutritional status in chronic obstructive pulmonary disease. Am. J. Respir. Crit. Care Med..

[24] Swallow E.B., Reyes D., Hopkinson N.S., Man W.D., Porcher R., Cetti E.J., Moore A.J., Moxham J., Polkey M.I. (2007). Quadriceps strength predicts mortality in patients with moderate to severe chronic obstructive pulmonary disease. Thorax.

[25] Després J.P., Lemieux I. (2006). Abdominal obesity and metabolic syndrome. Nature.

[26] van den Borst B., Gosker H.R., Koster A., Yu B., Kritchevsky S.B., Liu Y., Meibohm B., Rice T.B., Shlipak M., Yende S. (2012). The influence of abdominal visceral fat on inflammatory pathways and mortality risk in obstructive lung disease. Am. J. Clin. Nutr..

[27] McNicholas W.T. (2009). Chronic obstructive pulmonary disease and obstructive sleep apnea: Overlaps in pathophysiology, systemic inflammation, and cardiovascular disease. Am. J. Respir. Crit. Care Med..

[28] Gozal D., Kheirandish-Gozal L. (2008). Cardiovascular morbidity in obstructive sleep apnea: Oxidative stress, inflammation, and much more. Am. J. Respir. Crit. Care Med..

[29] Fletcher E.C., Schaal J.M., Miller J., Fletcher J.G. (1987). Long-term cardiopulmonary sequelae in patients with sleep apnea and chronic lung disease. Am. Rev. Respir. Dis..

[30] Somers V.K., White D.P., Amin R., Abraham W.T., Costa F., Culebras A., Daniels S., Floras J.S., Hunt C.E., Olson L.J. (2008). Sleep apnea and cardiovascular disease: An American Heart Association/American College of Cardiology Foundation Scientific Statement from the American Heart Association Council for High Blood Pressure Research Professional Education Committee, Council on Clinical Cardiology, Stroke Council, and Council on Cardiovascular Nursing. J. Am. Coll. Cardiol..

[31] Hawrylkiewicz I., Sliwinski P., Gorecka D., Plywaczewski R., Zielinski J. (2004). Monaldi Arch Chest Pulmonary haemodynamics in patients with OSAS or an overlap syndrome. Monaldi Arch. Chest Dis..

[32] Ganga H.V., Nair S.U., Puppala V.K., Miller W.L. (2013). Risk of new-onset atrial fibrillation in elderly patients with the overlap syndrome: A retrospective cohort study. J. Geriatr. Cardiol..

[33] Szathmary M., Sárközi H.K., Ianoși E.S., Gáll Z., Nemes A.F., Vultur M.A., Neagos A., Jimborean G. (2022). Súlyos krónikus obstruktív tüdőbetegségben szenvedő betegek tüdőfunkciója és társbetegségei a marosvásárhelyi Tüdőgyógyászati Klinikán. Orvosi Hetil..

[34] Muraki I., Wada H., Tanigawa T. (2018). Sleep apnea and type 2 diabetes. J. Diabetes Investig..

[35] Vale J., Manuel P., Oliveira E., Oliveira A.R., Silva E., Melo V., Sousa M., Alexandre J.C., Gil I., Sanchez A. (2015). Obstructive sleep apnea and diabetes mellitus. Rev. Port. Pneumol..

[36] Doumit J., Prasad B. (2016). Sleep Apnea in Type 2 Diabetes. Diabetes Spectr..

[37] Park S.S., Perez Perez J.L., Perez Gandara B., Agudelo C.W., Rodriguez Ortega R., Ahmed H., Garcia-Arcos I., McCarthy C., Geraghty P. (2022). Mechanisms Linking COPD to Type 1 and 2 Diabetes Mellitus: Is There a Relationship between Diabetes and COPD?. Medicina.

[38] Tufik S., Gozal D., Ishikura I.A., Pires G.N., Andersen M.L. (2020). Does obstructive sleep apnea lead to increased risk of COVID-19 infection and severity?. J. Clin. Sleep Med..

[39] Lugade A.A., Bogner P.N., Thatcher T.H., Sime P.J., Phipps R.P., Thanavala Y. (2014). Cigarette smoke exposure exacerbates lung inflammation and compromises immunity to bacterial infection. J. Immunol..

[40] Abe Y., Murphy T.F., Sethi S., Faden H.S., Dmochowski J., Harabuchi Y., Thanavala Y.M. (2002). Lymphocyte proliferative response to P6 of Haemophilus influenzae is associated with relative protection from exacerbations of chronic obstructive pulmonary disease. Am. J. Respir. Crit. Care Med..

[41] Szathmáry M., Gîrbovan E.-C., Sárközi H.-K., Gáll Z., Vultur M.A., Nemeș A.F., Ianoși E.S., Jimborean G. (2023). Obstruktív tüdőbetegségek súlyosbodása SARS-CoV-2-fertőzés hatására a marosvásárhelyi Pulmonológiai Klinika beteganyagában. Orvosi Hetil..

[42] Iasi R.S.C.C.E.H., Vultur M.A., Hédi-Katalin S., Ianosi M.B., Szathmary M., Ianosi E.S., Postolache P., Jimborean G. (2021). Association between diabetes mellitus and SARS-CoV2 infection—A retrospective study in the Pulmonology Clinic of Târgu Mureș. Med. Surg. J. Rev. Med. Chir. Soc. Med. Nat. Iasi.

[43] https://www.cdc.gov/obesity/data/obesity-and-covid-19.html.

[44] Tang M., Wang Y., Wang M., Tong R., Shi T. (2021). Risk for Cardiovascular Disease and One-Year Mortality in Patients With Chronic Obstructive Pulmonary Disease and Obstructive Sleep Apnea Syndrome Overlap Syndrome. Front. Pharmacol..

[45] Soler X., Diaz-Piedra C., Ries A.L. (2013). Pulmonary rehabilitation improves sleep quality in chronic lung disease. J. Chronic Obstr. Pulm. Dis..

[46] Venkatesan P. (2024). GOLD COPD report: 2024 update. Lancet Respir. Med..

[47] Braido F., Baiardini I., Cazzola M., Brusselle G., Marugo F., Canonica G.W. (2013). Long-acting bronchodilators improve Health Related Quality of Life in patients with COPD. Respir. Med..

[48] Sterling K.L., Pépin J.-L., Linde-Zwirble W., Chen J., Benjafield A.V., Cistulli P.A., Cole K.V., Emami H., Woodford C., Armitstead J.P. (2022). Impact of Positive Airway Pressure Therapy Adherence on Outcomes in Patients with Obstructive Sleep Apnea and Chronic Obstructive Pulmonary Disease. Am. J. Respir. Crit. Care Med..

[49] Borel J.-C., Tamisier R., Gonzalez-Bermejo J., Baguet J.-P., Monneret D., Arnol N., Roux-Lombard P., Wuyam B., Levy P., Pépin J.-L. (2012). Noninvasive ventilation in mild obesity hypoventilation syndrome: A randomized controlled trial. Chest.

[50] Tondo P., Scioscia G., Sabato R., Leccisotti R., Hoxhallari A., Sorangelo S., Mansueto G., Campanino T., Carone M., Barbaro M.P.F. (2023). Donato Lacedonia, Mortality in obstructive sleep apnea syndrome (OSAS) and overlap syndrome (OS): The role of nocturnal hypoxemia and CPAP compliance. Sleep Med..

[51] Adler D., Bailly S., Soccal P.M., Janssens J.P., Sapène M., Grillet Y., Stach B., Tamisier R., Pépin J.L. (2021). Symptomatic response to CPAP in obstructive sleep apnea versus COPD-obstructive sleep apnea overlap syndrome: Insights from a large national registry. PLoS ONE.

[52] Kapur V.K., Auckley D.H., Chowdhuri S., Kuhlmann D.C., Mehra R., Ramar K., Harrod C.G. (2017). Clinical Practice Guideline for Diagnostic Testing for Adult Obstructive Sleep Apnea: An American Academy of Sleep Medicine Clinical Practice Guideline. J. Clin. Sleep Med..

[53] Stanchina M.L., Welicky L.M., Donat W., Lee D., Corrao W., Malhotra A. (2013). Impact of CPAP use and age on mortality in patients with combined COPD and obstructive sleep apnea: The overlap syndrome Observational. Study J. Clin. Sleep Med..

[54] Wang Y., Su M., Zhang X. (2014). Effects of continuous positive airway pressure treatment of inflammatory factors in patients with overlap syndrome. Zhonghua Yi Xue Za Zhi.

[55] Nural S., Günay E., Halici B., Celik S., Ünlü M. (2013). Inflammatory processes and effects of continuous positive airway pressure (CPAP) in overlap syndrome. Inflammation.

[56] Crisafulli E., Barbeta E., Ielpo A., Torres A. (2018). Management of severe acute exacerbations of COPD: An updated narrative review. Multidiscip. Respir. Med..

[57] Piper A.J., Wang D., Yee B.J., Barnes D.J., Grunstein R.R. (2008). Randomised trial of CPAP vs bilevel support in the treatment of obesity hypoventilation syndrome without severe nocturnal desaturation. Thorax.

[58] Czerwaty K., Dżaman K., Sobczyk K.M., Sikorska K.I. (2022). The Overlap Syndrome of Obstructive Sleep Apnea and Chronic Obstructive Pulmonary Disease: A Systematic Review. Biomedicine.

[59] Zhang P., Chen B., Lou H., Zhu Y., Chen P., Dong Z., Zhu X., Li T., Lou P. (2022). Predictors and outcomes of obstructive sleep apnea in patients with chronic obstructive pulmonary disease in China. BMC Pulm. Med..

[60] Fanaridis M., Bouloukaki I., Stathakis G., Steiropoulos P., Tzanakis N., Moniaki V., Mavroudi E., Tsiligianni I., Schiza S. (2024). Prevalence and Characteristics of Patients with Obstructive Sleep Apnea and Chronic Obstructive Pulmonary Disease: Overlap Syndrome. Life.

[61] Srivali N., Thongprayoon C., Tangpanithandee S., Cheungpasitporn W., Won C. (2023). The use of continuous positive airway pressure in COPD-OSA overlap syndrome: A systematic review. Sleep Med..

[62] Voulgaris A., Archontogeorgis K., Steiropoulos P., Papanas N. (2021). Cardiovascular Disease in Patients with Chronic Obstructive Pulmonary Disease, Obstructive Sleep Apnoea Syndrome and Overlap Syndrome. Curr. Vasc. Pharmacol..

[63] Tang M., Long Y., Liu S., Yue X., Shi T. (2021). Prevalence of Cardiovascular Events and Their Risk Factors in Patients with Chronic Obstructive Pulmonary Disease and Obstructive Sleep Apnea Overlap Syndrome. Front. Cardiovasc. Med..

[64] Bouloukaki I., Fanaridis M., Testelmans D., Pataka A., Schiza S. (2022). Overlaps between obstructive sleep apnoea and other respiratory diseases, including COPD, asthma and interstitial lung disease. Breathe.

[65] van Zeller M., McNicholas W.T. (2024). Sleep disordered breathing: OSA-COPD overlap. Respir. Med..

[66] Suri T.M., Suri J.C. (2021). A review of therapies for the overlap syndrome of obstructivesleep apnea and chronic obstructive pulmonary disease. FASEB BioAdv..

